# Phosphocreatine recovery time constant (PCr) at peak exercise as a potential endpoint for clinical trials in PAD

**DOI:** 10.1186/1532-429X-18-S1-P352

**Published:** 2016-01-27

**Authors:** Jorge A Gonzalez, Yan Li, Peter W Shaw, Pelbreton Balfour, Yang Yang, Jennifer Kay, Joseph DiMaria, Arthur Weltman, Michael Salerno, Craig H Meyer, Frederick H Epstein, Christopher M Kramer

**Affiliations:** 1 grid.27755.32000000009136933XCardiology, University of Virginia, Charlottesville, VA USA; 2 grid.27755.32000000009136933XRadiology, University of Virginia, Charlottesville, VA USA; 3 grid.27755.32000000009136933XBiomedical Engineering, University of Virginia, Charlottesville, VA USA; 4 grid.27755.32000000009136933XMedicine, University of Virginia, Charlottesville, VA USA

## Background

Patients with PAD who have intermittent claudication have reduced exercise tolerance, however, the mechanisms are not well understood. Ankle-brachial index (ABI) correlates neither with walking distance nor the degree of claudication or functional limitation as it is only able to measure flow at the macrovascular level. ^31^phosphocreatine (^31p^) is a non-invasive marker of mitochondrial capacity in the skeletal muscle providing an insight into muscle metabolism and tissue perfusion. We sought to analyze the relationship of ^31p^ recovery time constant (PCr) and exercise capacity in a population with PAD.

## Methods

Twenty-three (23) patients with PAD (ABI < 0.9) were prospectively enrolled. All performed supine plantar flexion exercise at 50 rpm using a pedal ergometer until exhaustion or limiting symptoms. PCr was measured by ^31p^ MR spectroscopy using a 3 T Siemens Trio MR scanner during recovery after peak exercise. A single-pulse, surface coil localized 512 msec free induction decay acquisition with 20 averages centered on the midcalf was used. A standard ^31p^ surface coil in the patient table is employed. PCr was then calculated using a monoexponential fit of phosphocreatine concentration versus time, beginning at cessation of exercise. Patients later exercised on a treadmill (Gardner exercise protocol) and completed a 6 min-walk protocol. Peak VO_2_ (peak oxygen consumption) was measured. METS (Metabolic equivalents), total distance (feet), total exercise time and start of claudication time were also recorded.

## Results

The mean age was 67 ± 11 years, 63% were male, 63% were Caucasian, the mean ABI was 0.69 ± 0.09. The mean logPCr was 1.68 ± 0.25, 6-min walk distance 1024.7 ± 351.9 feet, 6-min walk start of claudication 330.0 ± 274.2 feet, peak VO_2_ 13.4 ± 3.7 (ml/kg/min), METS 3.8 ± 1.1, treadmill exercise time 5.8 ± 4.4 min. PCr correlated with 6-min walk total distance (Pearson's r: 0.43, p = 0.04), total METS (r = 0.49, p = 0.02) and peak VO_2_ (r = 0.49, p = 0.02). No correlation was seen between PCr and claudication distance (p = 0.27) nor start of leg discomfort (p = 0.24). ABI did not correlate with any of these exercise parameters.

## Conclusions

PCr recovery time constant correlates significantly with total distance, METS and peak VO_2_ in patients with PAD whereas ABI does not. PCr recovery kinetics could be used a therapeutic target in novel interventions in patients with PAD as it correlates better with exercise parameters than ABI.

**Figure 1:**
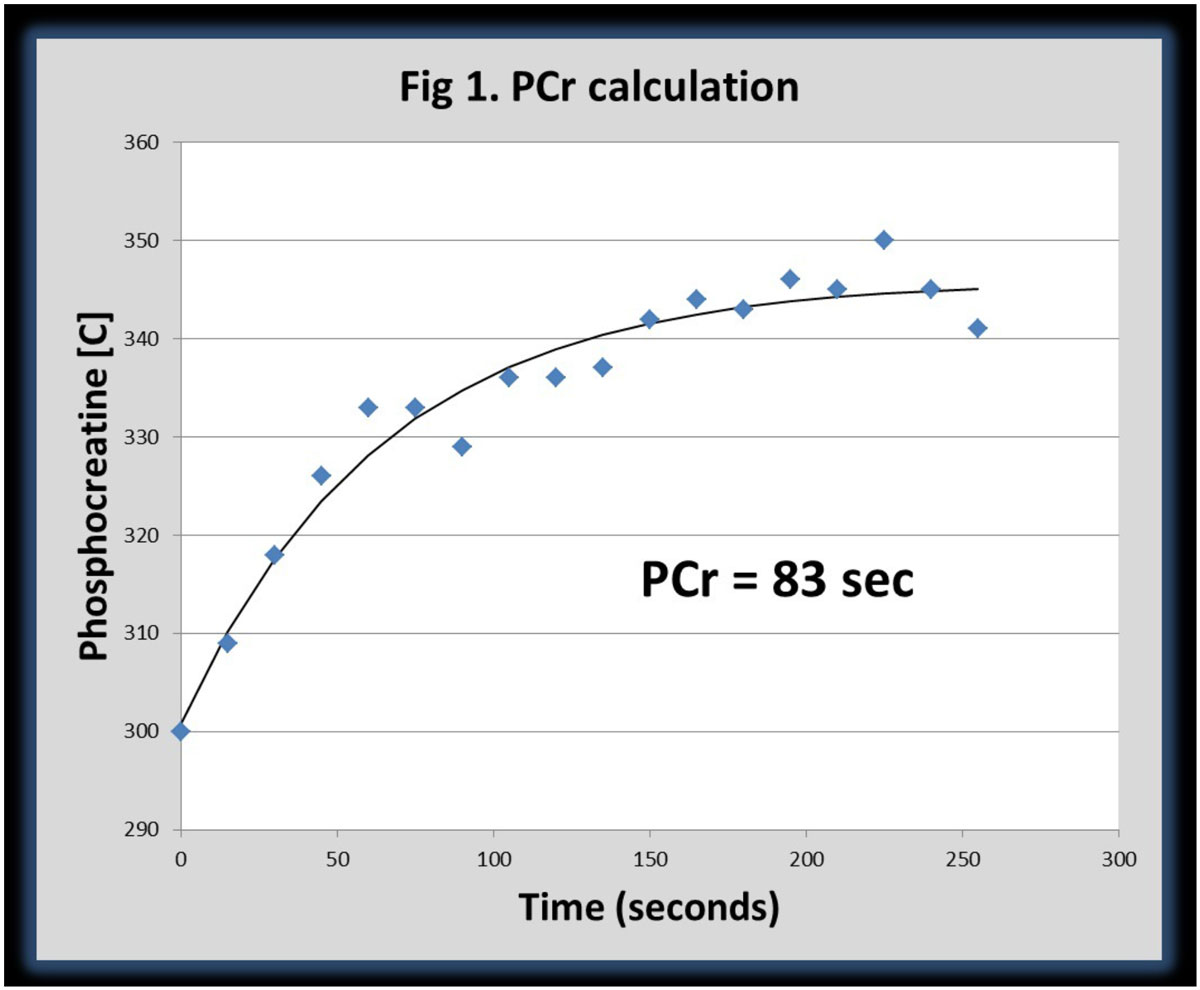
**PCr calculation based of the monoexponential fit of phosphocreatine concentration vs time**.

